# Phytochemical-Based Nano-Pharmacotherapeutics for Management of Burn Wound Healing

**DOI:** 10.3390/gels7040209

**Published:** 2021-11-13

**Authors:** Abdul Qadir, Samreen Jahan, Mohd Aqil, Musarrat Husain Warsi, Nabil A. Alhakamy, Mohamed A. Alfaleh, Nausheen Khan, Athar Ali

**Affiliations:** 1 Department of Pharmaceutics, School of Pharmaceutical Education & Research, Jamia Hamdard, New Delhi 110062, India; aqkhan90@gmail.com (A.Q.); samreenj1996@gmail.com (S.J.); aqilmalik@yahoo.com (M.A.); 2 Department of Pharmaceutics and Industrial Pharmacy, College of Pharmacy, Taif University, Al-Haweiah, Taif 21974, Saudi Arabia; 3 Department of Pharmaceutics, Faculty of Pharmacy, King Abdulaziz University, Jeddah 21589, Saudi Arabia; nalhakamy@kau.edu.sa (N.A.A.); maalfaleh@kau.edu.sa (M.A.A.); 4 Vaccines and Immunotherapy Unit, King Fahd Medical Research Center, King Abdulaziz University, Jeddah 21589, Saudi Arabia; 5 Department of Pharmacognosy and Phytochemistry, School of Pharmaceutical Education & Research, Jamia Hamdard, New Delhi 110062, India; nausheenkhan070@gmail.com; 6 Centre for Transgenic Plant Development, Department of Biotechnology, Jamia Hamdard, New Delhi 110062, India; atharbiotech@gmail.com

**Keywords:** burn, injury, phytochemical, nanotechnology, wound healing

## Abstract

Medicinal plants have been used since ancient times for their various therapeutic activities and are safer compared to modern medicines, especially when properly identifying and preparing them and choosing an adequate dose administration. The phytochemical compounds present in plants are progressively yielding evidence in modern drug delivery systems by treating various diseases like cancers, coronary heart disease, diabetes, high blood pressure, inflammation, microbial, viral and parasitic infections, psychotic diseases, spasmodic conditions, ulcers, etc. The phytochemical requires a rational approach to deliver the compounds to enhance the efficacy and to improve patients’ compatibility. Nanotechnology is emerging as one of the most promising strategies in disease control. Nano-formulations could target certain parts of the body and control drug release. Different studies report that phytochemical-loaded nano-formulations have been tested successfully both in vitro and in vivo for healing of skin wounds. The use of nano systems as drug carriers may reduce the toxicity and enhance the bioavailability of the incorporated drug. In this review, we focus on various nano-phytomedicines that have been used in treating skin burn wounds, and how both nanotechnology and phytochemicals are effective for treating skin burns.

## 1. Introduction

Skin is the largest visible and vulnerable organ of the human body. It protects our body from environmental changes and dehydration [1,2]. There are certain skin conditions, such as burns and other substantial loss of the outer layer of the skin (epidermis), which acts as the barricade that prevents the skin from degeneration and microbial incursion and balances the fluid levels of the body. In such conditions, both nutritional and electrolytes constituents get demolished. Hence, skin wounds can drastically impact human health [3]. Various diseases, such as eczema, herpes zoster, rosacea, and psoriasis, can cause harm to skin; however, burns are the major cause of skin damage [4]. According to the World Health Organization (WHO), an estimated 180,000 deaths are caused by burns annually [5]. A burn injury may result from hot and cold materials and vulnerability to chemicals and radiations. Burn wounds are of three types and classified by the profundity: (1) superficial (first degree), (2) partial thickness (second degree), and (3) full thickness (third degree) [6,7]. Healing of burn wounds is a complicated process, and it proceeds through various phases, including inflammation, proliferation, and remodeling. These phases should occur in the proper order and time sequence for better would healing as changes in any of the phases may cause a delay in the healing process [8,9]. After epidermal injury, platelet activation leads to control of blood loss and results in clot formation, which is the first step in wound healing mechanism [10]. Nanotechnology offers an excellent outlook to fast-track persistent wound healing by altering the different phases of healing with high payloads of phytoconstituents [11]. Over the last two centuries, the use of plants extracts in wound healing has increased due to the presence of active compounds in these extracts [12]. Herbal medicines are widely accepted because of their efficacy and low level of adverse effects [13]. For skin-related diseases and other disorders, utilization of herbal constituents is accepted by 80% of population [14]. Using different plant extracts, various studies have been performed to observe the pharmacological action of constituents on various disease. From 2011 to present day, the use of herbal medicines has increased from $18 million to $26 billion; it is also estimated that 50% of approved herbal drugs are provided worldwide [15,16].

Nanocarriers with herbal drugs have gathered significant recognition for their potential and distinctive attributes in numerous domains of human activity [17].

The combination of nanotechnology with natural drugs would be a novel development for enhancing the medicinal effect of these natural drugs [18]. To increase the acceptability of these compounds by patients and to prevent the need for repeated administration, the phytochemical needs an approach that can encourage the delivery of active components in a sustained release format. Novel drug delivery systems help achieve the required therapeutic effects with reduced adverse events and enhance the bioavailability of herbal constituents [19,20].

Comprehensive searches were done on Google Scholar and PubMed databases pertaining to herbal-based pharmaceuticals for burn wound, nano-drug delivery applications in burn healings, and past to present evolution of nano-phytomedicines for the management of burn wound healing. We focused mainly on the last 15 years of works in this area, although some older references were also included to provide validity to the review.

## 2. Wound Healing Process

**Figure 1:**
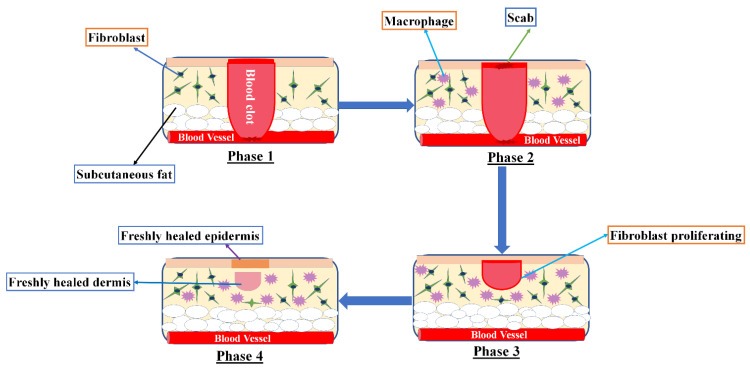
Different phases of the wound-healing process.

Would healing is a complicated process, and it progresses through various phases, including inflammation, proliferation, and remodeling. Involvement of fibroblasts, leukocytes, and monocytes in the healing process aid in reconstituting the destructed skin (Figure 1). Vitamins E and C play crucial roles in wound healing as these are key factors in this process. Vitamin K prevents severe bleeding, carotenoids restore the skin epithelial layer and tissues, and phytosterols have antimicrobial and anti-inflammatory effects [21]. Would healing also involves the use of biochemical genetic reprogramming to reinstate the skin health. Recent research has shown that the use of phytochemicals has active constituents that have the capability to induce wound healing with less side effects [22].

### 2.1. Inflammatory Phase

The inflammatory phase starts 4–5 days after the skin damage has already occurred. Once injury occurs, intravascular platelets mediate homeostasis, which is responsible for clot formation over the wound to stop further bleeding [23]. Post-homeostasis, thrombin activates platelets, which releases multiple growth factors, such as epidermal growth factor (EGF), insulin growth factor-1 (IGF-1), platelet derived growth factor (PDGF), fibroblast growth factor (FGF), and transforming growth factor (EGF, IGF-1, PDGF, FGF, and TGF, respectively) [24,25]. These growth factors activate neutrophils, monocytes, leukocytes, and macrophages, which act as a shield for skin to prevent further damage and initiate the wound healing process [26,27].

### 2.2. The Proliferative Phase

The proliferative phase involves cell proliferation and migration and takes 3–15 days to activate [28]. Once PDGF is released by platelets during the inflammatory phase, formation of new blood vessels and capillaries occurs [29]. Following this step, angiogenesis and migration of fibroblasts takes place to form granulation tissues [30]. Once the fibroblasts process is complete, a new extracellular matrix (ECM) consisting of collagen and proteoglycans is produced. Some fibroblasts undergo fission into myofibroblasts, which helps with the contraction of the wounded area [31]. Following this step, activation of keratinocytes, which migrate to the injured area and completes the last step, that of re-epithelialization, occurs [32].

### 2.3. Re-Modeling Stage

The re-modeling stage continues to change over the first several weeks to several years after the wound occurs. Collagen I replace collagen III, which consists of newly synthesized ECM, and the fresh collagen fibers develop into an assembled lattice composition that enhances the intensity of healed tissues [33,34].

## 3. The Impact of Antibiotics and Antioxidant Properties of Plants

Since ancient times, various plants have been used for treatment of different diseases and are currently in use worldwide. Due to their antimicrobial activities, natural constituents, such as aminoglycosides [35], beta-lactams [36], glycopeptides [37], quinolones [38], sulfonamides [39], and tetracyclines [40] have been utilized for wound healing treatment. For wound therapy, the benefits of plant extracts or phytochemical have been recognized as has the existence of antioxidants in numerous plants extracts. The presence of oxygen free radicals causes disruption in the wound healing process. Stress caused by oxidation slows down the healing process and causes additional damage to tissues. An antioxidant confers protection from oxygen free radicals by reducing their effects; this process assists in the wound healing process. Active compounds possessing antimicrobial effects play an important role as they neutralize free oxygen radicals and enhance the wound healing process [41,42]. Antioxidants have been used to hasten healing activity by extending antioxidant effects throughout the healing process. It seems the existence of antioxidants is essential to facilitate recuperation from persistent skin injury; Table 1 shows plants with both antimicrobial and antioxidant properties [43]. Natural metabolites influence the wound healing process by introducing various growth factors, such as EGF and FGF, which affect cellular movement [44]. Animal studies have indicated that herbal compounds encourage anti-inflammatory and antimicrobial activities for wound treatment by promoting regeneration of skin cells and displacing connective tissues [45]. Table 1 shows the names of natural compounds having antioxidant and antibiotic activities toward wound healing.

The efficacy of *Centella asiatica* has been studied broadly in animal models. This herb helps heal the incision-induced injuries. In one study, it was concluded that the level of antioxidants increases extensively in the presence of this herb, leading to improved healing activity [46]. Asiaticoside is extracted from *Centella asiatica* and produces a better capacity for injury healing process in both chronic and immediate healing as it has fibroblast proliferating activity [47].

Leaf extracts from *Chromolaena odorata* contain flavonoids and this herb has been used widely in the wound healing activity due to the free radical approach, which has shown a conclusive promotion in healing activity [48]. This extract was shown to cause improvement in the fibroblast proliferation and keratinocyte and endothelial cell activity, and to stimulate keratinocyte migration [48].

*Quercus infectoria* Olivier possesses anti-inflammatory, anti-bacterial, and antioxidant properties. The ethanolic extract of gallic acid, ellagic acid, and syringic acid form the active constituents of tannins, which might be the reason for antioxidant effects resulting in enhanced healing activity. In this study, the incision wound animal model showed better healing activity after stimulation with antioxidants, which caused enhancement of the superoxide dismutase and catalase levels, both of which are influential antioxidant enzymes [49].

Wounds, burns, and internal and external ulcers can be treated by *Buddleja globasa* (common name: orange bell Buddleja). This herb is also used traditionally in Chile for the treatment of ulcers and burns. This herb was tested for its capability to stimulate fibroblast growth and antioxidant activity in vitro. Testing was specific as the effect of the aqueous solution of *B. globasa* on these two processes is considered as the first stage in the tissue repair cycle [50]. It was proven that the damage caused by oxygen free radical causes a delay in the healing process; thus, an antioxidant was needed to reverse this delay [51]. This process was achieved because of the presence of flavonoids and caffeic acid in the extract. Buddleja leaves have other applications in the skin layer formation that is part of wound healing [52].

Curcumin is a derivative of *Curucuma longa*, which is also known as turmeric and Haldi. *C. longa* has numerous biological properties, which consist of antioxidant, initiation of enzyme detoxification, and prevention of degenerative diseases [53]. Dermal application of these substances to patients resulted in enhanced wound healing activity and provided tissue defense from oxidation-induced damage [54].

**Table 1:** Phytochemicals having antibiotic, antimicrobial, and antioxidants activity on wound healing.

Medicinal Plants	Family	Active Ingredients	Activities	Reference
*Acacia Senegal* L.	Leguminosae	Saponins, alkaloids, and malic acid	Antimicrobial, anti-inflammatory, and antioxidant activity	[55]
*Acalypha indica* L.	Euphorbiaceae	Flavanoids, alkaloids, saponins	Antioxidant and antimicrobial activity	[55]
*Allophylus rubifolius* L.	Sapindaceae	-	Antioxidant, antibacterial, and anti-inflammatory activity	[55]
*Anagallis arvensis* L.	Primulaceae	flavonoids, saponins, glycosides, alkaloids, and anthraquinones	Antioxidant, anti-inflammatory, and antimicrobial activity	[56]
*Anogeissus dhofarica* L.	Combretaceae	-	Antioxidant and antimicrobial activity	[41]
*Aloevera* L.	Liliacae	Saponins, acemannan, and anthraquinone	Antimicrobial activity	[57]
* Anethum graveolens * L.	Umbelliferae	-	Anti-inflammatory and antibacterial activity	[58]
*Aristolochia bracteolate* L.	Aristolochiaceae	-	Antimicrobial and antioxidant activity	[59]
*Alternanthera brasiliana* L.	Amaranthaceae	-	Antimicrobial activity	[60]
*Achillea millefolium* L.	Asteraceae	Isovaleric acid, salicylic acid, sterols, flavonoids, tannins, and coumarins	Antimicrobial activity	[15]
*Acanthus polystachyus* L.	Acanthaceae	Tannins, flavonoids, saponins, polyphenols, terpenoids, glycosides, and anthraquinones	Anti-inflammatory and antioxidant activity	[61]
*Becium dhofarense* L.	Lamiaceae	-	Antioxidant activity	[55]
*Bridelia ferruginea* L.	Phyllanthaceae	Flavonoids, tannins, saponins, and terpenoids	Antioxidant and antibacterial activity	[62]
*Buddleja globosa* L.	Scrophulariaceae	-	Antioxidant activity	[50]
*Centella asiatica* L.	Araliaceae	Flavonoids	Antioxidant activity	[46,63,64]
*Chromolaena odorata* L.	Asteraceae	Alkaloids, flavonoids, flavanone, essential oils, phenolics, saponins, tannins, and terpenoids	Antimicrobial, antioxidant, and anti-inflammatory activity	[48]
*Clerodendrum infortunatum* L.	Lamiaceae	Flavonoids	Antioxidant and antimicrobial activity	[65]
*Combretum smeathmanii* L.	Combretaceae	Alkaloids, coumarins, flavonoids, saponins, terpenes, and sterols	Antioxidant and antimicrobial activity	[66]
*Cordia perrottettii* L.	Boraginaceae	-	Antioxidant activity	[55]
*Curcuma longa* L.	Zingiberaceae	Glycosides, tannins, and flavonoids	Anti-inflammatory, antimicrobial, and antioxidant activity	[53,54,67]
*Crassocephalum crepidioides* L.	Asteraceae	Phenolic, flavonoid, and essential oil	Anti-inflammatory and antioxidant activity	[68]
*Cinnamomum verum* L.	Lauraceae	Tannins	Anti-inflammatory, antimicrobial, and antioxidant activity	[69]
*Dendrophthoe falcata* L.	Loranthaceae	-	Antioxidant and antimicrobial activity	[70]
*Eucalyptus globulus* L.	Myrtaceae	Alkaloids, flavonoids, saponin, tannin, carbohydrates, and glycosides etc.	Anti-inflammatory activity	[71]
*Ficus asperifolia* L.	Moraceae	Flavonoids, phenolics, alkaloids, and tannins	Antioxidant and antimicrobial activity	[72]
*Gossypium arboreum* L.	Malvaceae	Alkaloids, phenolic compounds, terpenoids, tannins, saponins flavonoids, cardiac glycosides, and protein	Antioxidant and antimicrobial activity	[72]
*Gunnera perpensa* L.	Gunneraceae	Alkaloids, ellagic acids, flavonoids, phenols, benzoquinones, proanthocyanidins, tannins, and minerals	Anti-inflammatory, antioxidant, and antibacterial activity	[73]
*Hippophae rhamnoides* L.	Elaeagnaceae	Flavonoids, tannins, triterpenes, glycerides of palmitic, stearic, oleic acids, vitamins (C, E, K), and amino acids	Anti-inflammatory, antimicrobial, and antioxidant activity	[74]
*Holoptelea integrifolia* L.	Ulmaceae	Terpenoids, saponins, tannins, phenols, alkaloids, flavonoids, glycosides, and quinines	Anti-inflammatory, antibacterial, and antioxidant activity	[75]
*Memecylon edule* L.	Melastomataceae	-	Anti-inflammatory activity	[76]
*Moringa peregrina* L.	Moringaceae	Proteins, vitamins, beta-carotene, amino acid, and phenolics	Anti-inflammatory, antimicrobial and antioxidant activity	[53,77]
*Olea europaea* L.	Oleaceae	Flavonoids, iridoids, secoiridoids, flavanones, biophenols, triterpenes, benzoic acid	Antioxidant activity	[53,78]
*Phyllanthus muellerianus* L.	Phyllanthaceae	Isoquercitrin, rutin, astragalin, phaselic acid, gallic acid, caffeic acid, methylgallate	Anti-inflammatory and antioxidant activity	[71]
*Plagiochasma appendiculatum* L.	Aytoniaceae	-	Antioxidant and antimicrobial activity	[79]
*Pluchea Arabica* L.	Asteraceae	Anthocyanins, phenolic acids, flavonoids, and carotenoids	Antioxidant activity	[53]
*Quercus infectoria* L.	Fagaceae	Tannin	Anti-inflammatory and antibacterial	[49]
*Rhizophora mangle* L.	Rhizophoraceae	Triterpenes, tannins and their glycosides	Antimicrobial and antioxidant activity	[80]
*Secamone afzelii* L.	Apocynaceae	alkaloids, tannins, cardiac glycosides and saponins	Antioxidant activity	[50]
*Trigonella foenum* L.	Papilionaceae	Carbohydrates, proteins, lipids, fibers, flavonoids, alkaloids, and saponins	Anti-inflammatory and antioxidant activity	[81]

## 4. Nanotechnology Involvement in Wound Healing Enhancement

**Figure 2:**
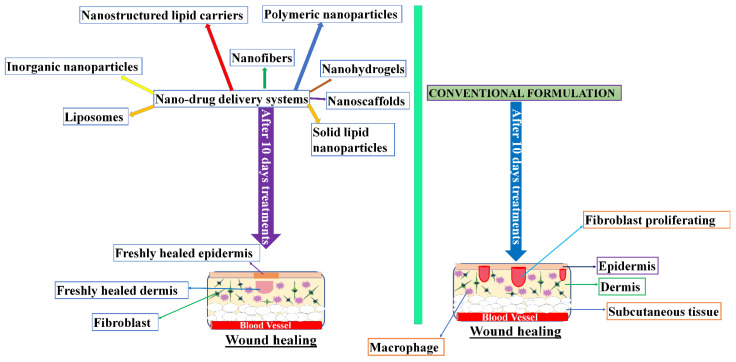
A comparison of nano-drug delivery systems involved in the skin regeneration and wound treatment with conventional drug delivery systems.

Nano-drug delivery systems enormously influence the potential of drugs’ medicinal effects and also protect the drugs from deterioration. Wound healing and skin re-formation involves various nano-delivery systems, such as those contained in organic nanoparticles, lipid nanoparticles, liposomes, polymeric nanoparticles, nanohydrogels, and nanofibers. These nano-systems show better efficacy compared to conventional systems (Figure 2).

In the past decades, a steady increase in the filing of patents based on herbal nano-formulations has been recorded. The key factor behind this increase is the capability of nano-formulations to overcome solubility drawbacks and bioavailability problems faced by conventional systems. One of most frequently filed patents is for curcumin, the multifunctional phytoceutical, extensively used in the treatment of tumors, cancers, and skin disorders. Other herbal based nano-formulations patents include carotenoids (nano-particles), silymarin (nano-particles), Panax ginseng (liquid mixture), *Syzygium cumini*, *Tinospora cordifolia*, *Trigonella foenum*-graecum, *Withania somnifera* (nano-emulsion, nanoencapsulation, nano-dispersion, or synergistic liquid mixture), and Arbutin (emulsified nanoparticles) [82].

### 4.1. Nanofibers

Nanofibers are formed by unbreakable polymer chains of natural and synthetic compounds, which act as a sheet of nanofibers when placed on the skin to improve the tissues [83]. Nanofibers imitate collagen fibrils in the ECM, which can be formed from the synthetic or natural compounds and have numerous qualities that provide benefits to the wound healing process [83]. Nanofibers are beneficial for wound healing because they have a permeable construction and great orifice connection. Nanofibers have the capability to keep moisture at a suitable level. The synthesis of nanofibers with phytochemicals in nanofibrous materials has yielded tremendous results in the area of wound healing as these fibers have the capability to reduce the incision mark because of their porosity, which allows movement of oxygen [84]. Emodin (1,3,8-trihydroxy-6-methyl-anthraquinone) is an anthraquinone derivative that is found in the roots of *Rheum officinale* L. and is used extensively for wound healing as it has antimicrobial and anti-inflammatory activities. It produced a positive result when used for acute skin injuries [85]. The nanofibers of emodin in polyvinylpyrrolidone were harmless, anti-allergenic, bioactive, and dissolved at a rapid rate when compared to the pure compound. Re-epitheliazation was shown to have occurred at the wounded area, which hastened the healing process [86]. To increase the composition of collagen in human cells to 100%, emodin was incorporated in cellulose acetate nanostructure fibers [87]. The development of herbal constituents in cellulose acetate nanofibers promotes wound healing by using biomaterials as an interactive dressing material. Asiaticoside is extracted from *C. asiatica*, and the incorporation of trisachharide triterpene into cellulose acetate nanofibers produces an antioxidative effect during the early stages of the injury healing [88]. Increases in types I and III pro-collagen mRNAs were shown to enhance skin fibroblasts by elevating the protein levels [89]. Curcumin incorporated into cellulose acetate caused an improvement in fibroblast proliferation, enhanced collagen synthesis, and protected the dermal fibroblast cells from oxidative stress caused by hydrogen peroxide (H_2_O_2_) [90].

The active constituent of turmeric curcumin (1,7-bis (4-hydroxy-3-methoxyphenyl)-1,6-heptadiene-3,5-dione) is a polyphenolic compound, which is obtained from *C. longa* L. Curcumin is an active ingredient and its use is widely accepted for wound healing because it possesses various properties, such as anti-inflammatory, antibacterial, and antioxidant ones [91]. For epidermal injury healing, curcumin has been used in various in-vivo animal models [92]. Re-epithelization occurs during the early stages and enhances coagulation synthesis because it releases TGFβ1, which leads to increases in the number of blood vessels and cell granulation [93]. Liakos et al. [94] suggested that essential oils, such as cinnamon, lemongrass, and peppermint, can be used as antimicrobial agents. These electro-spun cellulose-based nanofibrous dressings were shown to prevent the *Escherichia coli* growth and required lesser quantities of oils. These dressings did not show any kind of cytotoxic effects and appears to be safe to use.

It has been reported that the curcumin loaded poly(ε-caprolactone)/gum tragacanth (PCL/GT) led to an improvement in the mechanical properties and tensile strengths of nanofibers and had a positive impact on collagen content for the treatment of diabetic wounds. By 15 days after injury, this moiety led to rapid wound healing by causing regeneration of the epithelial layer [95]. Bromelain-loaded chitosan nanofibers produced favorable wound healing results. It was observed for the second degree burn and has positive impact. The chitosan 2% *w*/*v* bromelain showed better physiochemical results compared to chitosan 4% *w*/*v* bromelain and was effective in reducing burn-induced injuries [96]. Bixin-loaded polycaprolactone (PCL) nanofibers maintained and accelerated wound healing activity in excisional wounds and effectively reduced the scar tissue area on the diabetic mice [97]. Alfalfa nanofibers yielded better results with respect to skin regeneration as these nanofibers possess antibacterial activity and bioactive phytoestrogens that work as a building block for the dressings for regenerative wounds [98].

### 4.2. Polymeric Nanoparticles (PNPs)

Polymeric nanoparticles are biocompatible colloidal systems that have risen in importance for both biomedical and bioengineering applications [99]. They are generally integrated by charged polymers and connected by interactivity of cationic and anionic chains of groups [100]. When drugs are incorporated into polymeric systems, this process prevents the deterioration caused by proteases found in the injury and delivered in stages to lower the frequency of administration [101]. Polymeric nanomaterials are widely utilized because of their antibacterial and wound healing activities [102]. For re-generation of skin injury, keratinocyte growth factor (KGF) is an impressive and potent growth factor [103]. It was observed that the KGF consists of self-assembled nanovesicles that enhances healing of the injured tissue cells of the skin by enhancing epithelization and skin re-modeling [104,105]. Recently, PNPs have been formulated by poly-lactic-co-glycolic acid (PLGA) and some of the other combinations in polymeric systems, including alginate, gelatin, and chitosan [106]. PLGA is approved by the Food and Drug Administration (FDA) for use in PNPs. The size of the PGLA NPS is 1–200 nm, which provides the benefits of biodegradability, biocompatibility, and being innocuous [107]. PLGA particles are generally formulated by emulsification of lipophilic compounds utilizing numerous surfactants and organic solvents [108]. The development of EGF-loaded nanoparticles for injury healing using PGLA yielded a positive response with respect to fibroblast proliferation and enhancement of the healing activity in the full thickness wounded skin. EGF plays an importance role in mediating the de-differentiation of keratinocytes into an epithelial linage and to reestablishing the epithelial barrier [109]. One of the studies also suggested that PGLA might produce a biocompatible system for growth factor delivery. To reduce lactate levels and enhance wound healing activity, the peptide defense host, known as LL37, was incorporated into PGLA nanoparticles [110]. Natural polymers, such as chitosan, have been chiefly considered for wound healing activity because of antibacterial and biocompatibility activities [111]. Chitosan is cationic in nature and has been utilized for the inhibition of microbial-induced infections [112]. Chitosan nanovesicles (150–300 nm) are generally formulated utilizing the method of ionic gelation [113]. Nowadays, chitosan is widely accepted in wound treatment, and it can also be utilized as a prophylactic agent to inhibit the infection development and enhance healing activity [114,115]. Studies have shown that polylactic acid-loaded chitosan magnetic eugenol nanospheres had improved prevention and development of biofilm compared to pure chitosan, whilst performing endothelial proliferation [116]. Most of the studies concerning this topic have reported that nanovesicles containing chitosan and analogs might enhance healing activity by improving inflammatory cell function and restoring fibroblasts and osteoblast functions [117]. In two different studies, it was observed that chitosan-loaded nanovesicles improved the coagulation by binding to red blood cells (RBC) and ameliorating the function of inflammatory cells. In another study, the chitosan nanovesicles were used as the compounds in bandages outlined for the skin wound and, hence, enhanced healing activity in both humans and animals [115,118].

### 4.3. Dendrimers

Dendrimers are nanoscale (1–10 nm) systems with homogeneous structures that are monodispersed in polymer macromolecule that can be used for both therapeutic and diagnostic purposes. Subunits of phenyl acetylene were used to develop dendrimers [119,120]. In addition, functional groups present on the surface of dendrimers can operate as antibacterial agents. Dendrimers cause detachment of contaminated tissues and may extend the phase of inflammation and slow injury diminution in addition to promoting re-epithelization and better wound healing activity [121]. The interaction between positively and negatively charged groups present on dendrimers and on the bacterial cell wall would lead to the bacterial structure disturbance [121]. In another study, silver-loaded dendrimer NSs were observed to show anti-inflammatory and anti-microbial activities in a synergistic manner. These properties were also shown to prevent inflammation and enhance healing activity [122].

### 4.4. Metallic Nanoparticles

Metal-based nanoparticles are widely utilized as they produce antibacterial, antimicrobial, and anti-inflammatory effects. The chemical and physical structures of nanoparticles are important for determining the propensity of a nanoparticle to enter and/or bind to target cells with the capacity to interact with their biological machinery and elicit a response. The metal-based nanoparticles are widely accepted in medicine, and the most acceptable metallic nanoparticles are silver- and gold-based nanostructures. Herbal plants are widely accepted in the development of metallic nanoparticles because of their low levels of side effects and more therapeutic effects as compared to the conventional dosage form [123]. Most of the herbal extracts, such as *Cladophora fascicularis* [124], *Aerva lanata* [125], *Hippophae rhamnoides* [126], *Eucommia ulmoides* [127], Black tea leaf [128], *Averrhoa bilimbi* [129], *Salicornia brachiate* [130], *Abelmoschus esculentus* [131], olive leaf [132], *Ipomoea carnea* [133], *geranium* [134], and *Cissus arnotiana* [135] have been incorporated into metallic nanoparticles

Silver nanoparticles are widely used as they possess antimicrobial, antibacterial, and anti-inflammatory properties [136]. The solubility and bioactivity of the silver particles at the wounded area depend on the size of silver particles; the smaller the size is, the stronger the contact with the will skin be. Silver nanoparticle vesicle sizes range from 1 to 100 nm. In one study, the silver–silver chloride nanoparticles combined with lower grapheme oxide nanovesicles induced an escalation of the healing process because it generated a higher number of oxygen free radicals rather than free the silver ions. A positive impact on the antibacterial activity on both Gram-negative and -positive bacteria has been shown, and, hence, these particles can enhance wound healing activity as shown in in-vivo studies in mice [137]. ACTICOAT is an alternate form of silver antimicrobial barrier wound dressing, which prevents the complication of prior agents. It slows down the bacterial activity, which leads to a reduction in inflammation and causes an improvement in the healing process [138]. The plant-based bio-prepared nanoparticles reveal potential for wound remedy and bacterial infection prevention [139]. Different methods for the preparation of silver nanoparticles are used. Photochemical and chemical reduction are the two most widely used methods [140]. Different plant extracts have been incorporated into silver nanoparticles for wound healing containing alkaloids, glycoside, corticosteroids and essential oils [141]. *Cassia roxburghii* prepared silver nanoparticles show the potential for wound healing enhancement as these particles have significant antibacterial and antifungal activities [142].

The active constituent of *Drosera binata* is naphthoquinones, primarily plumbagin. *D. binata* silver nanoparticles show better antibacterial activity against *Staphylococcus aureus* without affecting human keratinocytes. It was also inconclusive as to whether it is *D. binata* extract or its pure form (3-chloroplumbagin) that would have effective results for antibiotics and, hence, enhance wound healing [143]. Extracts of grape pomace were also combined with silver nitrate, and grape-silver nanoparticle-stabilized liposomes were developed by Castangia et al. The resulting nano-formulation showed potential to offer a significant shield of keratinocytes and fibroblasts to combat oxidative stress, thus, avoiding cell damage and death [144]. The other highly acceptable nanoparticles in different applications, such as wound treatment, re-epithelization, and particularly drug delivery, include gold nanoparticles [145].

Their chemical stability and capability to absorb near-infrared (NIR) light combined with their positive impact and antibacterial activity will strengthen the wound healing process [146]. Gold nanoparticles have the potential to penetrate bacterial tissues and cause alterations in the cell membrane, which causes inhibition of bacterial activity [147], and also prevents bacteria from developing reactive oxygen species [148].

Gold nanoparticles are synthesized with collagen, gelatin, and chitosan to yield effective injury recovery activity and also helps to achieve the biocompatibility [149]. Chitosan-loaded gold nanoparticles showed enhanced results in the healing process as these particles increase free radical scavenging and improve biocompatibility; in the model, these particles enhance the formation of cells and lead to an improvement in hemostasis by increasing the healing activity in comparison to pure chitosan [150]. The resulting metabolites from *Indigofera aspalathoides* Vahl. (Papilionaceae), which is also known as Shivanarvembu, are extracted from plants and used for wound healing. The histopathology results demonstrate that the *I. aspalathoides* silver nanoparticles have a better effect on wound healing in mice. When treated with plant extract, the granulation tissue which possesses fibroblasts, collagen fibers, minimal edema, and newly developed blood vessels were noted [151]. The other forms of metallic nanoparticles are gold and copper oxide nanovesicles that improve wound healing, which leads to fast injury healing and slows down the infection development. Both silver and gold nanoparticles are formed by incorporating *Coleous forskohlii* root extracts. These particles exhibit antimicrobial activity and antioxidant activities and have a positive effect on re-epithelization at the site of wound, which enhances connective tissue formation and causes an increase in proliferation and remodeling rates of dermal cells [152]. The development of both titanium dioxide and copper oxide nanoparticles of *Moringa oleifera* and *Ficus religiosa* leaf extracts, respectively, were shown to enhance wound healing and decrease the removal wound site in rats [153].

### 4.5. Nanohydrogels

For wound treatment, nanohydrogels are considered to be effective carriers as they possess three-dimensional polymeric networks. Due to their permeable network, they have the capability to absorb the liquid, which helps the wound to keep hydrated and enhance the wound healing process by keeping the proper oxygen level. Due to their effectiveness, compatibility, and showing beneficial results on skin revitalization, nanohydrogels have become widely accepted [154].

To improve wound healing activity, the gellan cholesterol nanohydrogel is immersed in baicalin. The baicalin-loaded nanohydrogels manifest ideal efficacy for skin repair and also act as inflammation inhibitors when applied to an epidermal inflammation mice model in in-vivo studies [155]. The freshly developed nanocrystal bacterial cellulose hydrogels instantly stick to fibroblasts, support human dermal fibroblast morphology, restrict the relocation of cells, enhance the proliferation of cells, and influence the nine expressions of genes connected to healing of injury. These genes include interleukins 6 and 10, granulocyte-macrophage colony-stimulating factor, matrix metalloproteinase 2 (IL-6 and -10, GM-CSF, MMP-2, respectively), and TGF-β; hence, nanohydrogels play an important role in skin regeneration [156].

### 4.6. Liposomes

Liposomes appear to be an important vehicle for topical delivery; they are harmless and environmentally safe and possess high drug loading efficiency, long-term stability, biological acceptability with skin in addition to having the capability to incorporate both hydrophobic and hydrophilic drugs in water and bilayer cavities [157]. Liposomes successfully shield the injury site and build a humid habitat at the site of injury, which is beneficial for the healing of the wounded skin. Taking all these characteristics into consideration, liposomes have become widely accepted in skin regeneration and injury treatment [158]. A study on propylene glycol liposome nanocarriers demonstrated numerous merits in comparison to other nano-systems. This system showed the tendency to enhance the stability, retention, and permeation in the tissues of skin [159]. It surmised that propylene glycol ameliorate the elasticity of vesicle containing bilayer of phospholipids. Hence, it improved the permeation into the skin. Moreover, the particles size of liposomes should be 150 nm for better drug perforation into the skin layers [160]. Liposomes with silk fibroin hydrogels were prepared to stabilize the basic fibroblast growth factor (bFGF) that maintained the activity of proliferation of cells on wound fluids; it also enhances the healing process by inspiring angiogenesis [161]. Rabelo et al. assessed the gelatin-membrane consisting of usnic acid-loaded liposomes and obtained encouraging results for wound healing. These results showed that the membrane of liposomes prominently manages the second-grade infection on porcine model [162]. Furthermore, with improved collagen, accumulation on cellularized granulation tissue was discovered in the treated group of liposomal membrane, which when compared to one of the commercial products improved the granulation tissue maturation and repaired the scars [163]. Argan-liposomes and argan-hyalurosomes have been successfully developed by incorporating neem oil into them. These formulations were extremely biocompatible and could protect skin cells from oxidative stress effectively with improved efficacy of oil. Moreover, formulations stimulate wound closure substantially more effectively than oil dispersion [164]. The efficacy of mangiferin (employed in cure of skin lesions) was enhanced by modifying transferosomes with propylene glycol and glycerol. Improved deposition of mangiferin was observed in epidermal and dermal layer and fibroblasts were protected from oxidative stress and intensified their propagation [165].

### 4.7. Inorganic Nanoparticles

Inorganic nanoparticles are those derived from the inorganic materials and include carbon-, metal-, and ceramic-based nanovesicles that accelerate tissue repair and re-modeling. These particles deliver assistance in the region of medicines, counting cancer, imaging, and drug delivery; however, their utilization in tissue regulation and skin re-modeling is new, it also provides adhesion in tissue and enhanced antimicrobial activity in injury healing [166].

### 4.8. Lipid Nanoparticles

Lipid nanoparticles were designed to overcome the stability limitation of liposomes due to the lipid bilayer. Lipid nanovesicles consist of two types: (1) solid lipid nanoparticles (SLNs) and (2) nanostructured lipid carriers (NLCs). The preparation of lipid nanovesicles amid lipids molecules does not include the use of any potentially harmful biotic solvents [167]. In a study, both SLN- and NLC-loaded rh-EGF (epidermal growth factor) for chronic injury treatment were formulated by the emulsification followed by an ultrasonication method; however, the NLC process included no organic solvent and showed better entrapment efficiency. The results of both formulations show capabilities to enhance cell proliferation when compared with free rh-EGF and considerably enhance the healing activity for wound closure, re-establish the process of inflammation, and facilitate re-epithelization [168].

In another study, development of SLNs with the elastase inhibitor serpin A1 and antimicrobial peptide LL37 had a synergistic impact on injury healing. SLNs promoted the closure of injury in cells of fibroblasts and keratinocytes. Moreover, it also led to improvement in the activity of antibacterial against *S. aureus* and *E. coli* when compared with the LL37- and A1-treated groups [169].

## 5. Future Perspective and Conclusions

The main aim of this review article was to describe the advantages of using nano-systems for use in the wound healing process. The distinctive physiochemical properties of nano-systems make them a perfect candidate for the application of wound healing process. The wound therapy process by nanotechnological systems demonstrates better therapeutic effect compared to the conventional therapy for wound healing. Nanotechnological systems can change one or more than one phase of wound healing during the process, as it possesses antibacterial, anti-inflammatory, and anti-proliferation activities. Worldwide, the research has been conducted on natural and herbal compounds due to their more therapeutic effects and lesser side effects. There is a need for the development of improved systems for the delivery of drugs at the target site with a dose that does not alter the existing treatment of disease. The herbal compounds have great potential and, hence, a better future, especially when incorporated into the nanocarriers for chronic wound treatment as they have shown promising results. Herbal medicine-based novel drug delivery systems have acknowledged the approaches in the field of pharmaceuticals, which will improve the health of the people. It is also concluded that the incorporation of herbal compound in the nano-vehicle will aggrandize the magnitude of the existing delivery system. Anyhow, various approaches have been employed for the privileged application of nanocarriers in wound healing therapy. The main concerns for the nano-vehicles are toxicity because they may cause possible side effects in the human body. Hence, this requires to be rectified at the starting point for further progression of wound healing therapies in clinical trials. In in vivo models, there is slighter comprehension regarding non-material mediated wound healing processes and this is one of the problems observed. The studies of non-material-wound healing processes are based on in-vitro studies or mainly depend on single aim bacteria. The in-vivo wound healing application is required for the in-depth studies utilizing both Gram-positive and -negative bacterial strains. Subsequently, the main focus should be on improving and enhancing target efficiency for more efficacious wound healing. Therefore, the investigators should target producing a nanomaterial that is biocompatible and biodegradable and has the capability to correct all the phases of the wound healing process.

## Data Availability

Not applicable.
